# Future Care Plan at the Contemplation Stage Among Older Adults

**DOI:** 10.1093/geroni/igaa057.1215

**Published:** 2020-12-16

**Authors:** Abolade Oladimeji, Eva Kahana, Boaz Kahana, Jeongeun Lee

**Affiliations:** 1 case western reserve university, cleveland, Ohio, United States; 2 Case Western Reserve University, Cleveland, Ohio, United States; 3 Cleveland State University, cleveland, Ohio, United States; 4 Iowa State University, Iowa City, Iowa, United States

## Abstract

Previous studies have shown that making future care planning often involves several steps. This is consistent with the transtheoretical model of behavior change which identified stages in which behavior changes over time and progresses through a series of six stages (Prochaska & Norcross, 2001). Although most studies on future care planning among older adults have examined the progression from the pre-contemplation stage to an action stage, little is known about the unique characteristics of older adults at the contemplation stage. Our sample included 409 randomly selected older adults in Cleveland, Ohio who were in the first wave of a longitudinal study of successful aging (µage = 78.8, SD=5.74). We used multivariate logistic regression to predict older adults who are more likely to have thought of their future care plans (contemplation stage). Results indicate several individual and relationship factors such as marital status, engaging in health behaviors (not smoking), being young old, and having personal transportation (OR=.42, .63, 2.81, and 1.73) account for contemplating future care. These findings underscore that several individual resources, as well as relationship status, are associated with contemplation stage of future care planning. The results reinforce the idea that future care planning is a stage-based behavior that people engage in, rather than a stand-alone activity.

